# Standards for writing Society for Cardiovascular Magnetic Resonance (SCMR) endorsed guidelines, expert consensus, and recommendations: a report of the publications committee

**DOI:** 10.1186/s12968-021-00801-9

**Published:** 2021-11-04

**Authors:** Seth Uretsky, Niti Aggarwal, Ruud B. van Heeswijk, Saurabh Rajpal, Ethan Rowin, Michael D. Taylor, Johan W. Verjans, Anita Wokhlu, Michael Markl, Subha V. Raman, Dipan J. Shah

**Affiliations:** 1 Department of Cardiovascular Medicine, Gagnon Cardiovascular Institute, Morristown Medical Center/Atlantic Health System, Morristown, NJ USA; 2 Mayo Clinic, Rochester, MN USA; 3 Department of Radiology, Lausanne University Hospital (CHUV) and University of Lausanne (UNIL), Lausanne, Switzerland; 4 Division of Cardiology, Department of Internal Medicine, The Ohio State University, Columbus, OH USA; 5 CardioVascular Center, Tufts Medical Center, Boston, MA USA; 6 Cincinnati Children’s Hospital Medical Center, Cincinnati, OH USA; 7 Royal Adelaide Hospital, University of Adelaide, Adelaide, Australia; 8 Department of Medicine, Division of Cardiology, University of Florida, Gainesville, FL USA; 9 Departments of Radiology and Biomedical Engineering, Northwestern University, Feinberg School of Medicine and McCormick School of Engineering, Evanston, USA; 10 Indiana University and IU Health, Indianapolis, IN USA; 11 Houston Methodist DeBakey Heart and Vascular Center, Houston, TX USA

## Introduction

### Function of the publication committee

The field of cardiovascular magnetic resonance (CMR) is dynamic; CMR not only has undergone rapid scientific growth, but also is increasingly valued for its unique role in clinical cardiovascular (CV) care. The mission statement of the Society for Cardiovascular Magnetic Resonance (SCMR) is to improve cardiovascular health through advancing the field of CMR. Clinical excellence, education, and research are fundamental to this mission. Therefore, the SCMR is committed to facilitating the publication of clinical documents that promote the standards of best practice and dissemination of clinically relevant advances in the field of CMR. Oversight for the publication process is accomplished through the SCMR Publications Committee and Board of Trustees (BOT). The main role of the Publications Committee is to ensure that SCMR-endorsed publications are comprehensive, evidence-based, timely, and without bias, ensuring the highest clinical standards and scientific rigor. Similarly, a secondary role of the SCMR Publications Committee is to collaborate on joint recommendations or clinical documents with complementary CV imaging societies as well as the multiple stakeholders in CV care, including CV, medical, surgical, and radiology societies, associations, working groups, or special interest groups. Such joint statements often provide guidance pertaining to multimodality CV imaging or interdisciplinary standards of CV care.

In order to facilitate the publication of SCMR-endorsed documents, the publications committee performs the following specific activities: (1) soliciting and reviewing proposals for SCMR-endorsed publications from the SCMR leadership and its membership, (2) suggesting issues of importance for publication and recommending task groups to the SCMR Executive Committee, (3) making efforts to ensure that such task groups adequately represent the international membership of the SCMR in order limit bias and promote diversity, (4) broadly overseeing the writing process to ensure timely, state-of-the-art, and scientifically accurate communication that adequately meets the needs of CMR practitioners, (5) collaborating with other professional societies or joint task forces where appropriate, and (6) communicating with SCMR staff and the editorial staff of various peer-reviewed journals including SCMR’s own journal, the *Journal of Cardiovascular Magnetic Resonance [JCMR]*.

### Types of documents

As a result of these activities, to date, the SCMR has independently authored, endorsed, or jointly collaborated on over 35 publications. The types of papers that the publications committee can facilitate include:

A. Clinical practice guidelines or expert consensus statements on standardized CMR imaging, reporting, interpretation, indications, and patient-related issues including safety
B. Position statements as well as white papers on new or emerging CMR technologies, contrast agents, safety, policy, or standards or practices
C. Appropriate use criteria for CMR imaging, especially those specific to particular disease states or in the context of other imaging modalities and diagnostic tests

A *clinical practice guideline* is an evidence-based document meant to improve clinical outcomes and promote efficiency of care by identifying best practices and reducing practice variations. Such guidelines serve as a practical guide to physicians and other practitioners. They provide decision-making algorithms or recommendations based on systematic review, with attention to the strength of evidence and meta-analysis (if possible). They require synthesis of opinion from a diverse panel of experts. In order for practice guidelines to provide definitive and reliable recommendations, sufficient literature should be available to perform a comprehensive systematic review and meta-analysis. Such guidelines could have a clinical or technical focus.

An *expert consensus statement* is also meant to provide guidance for clinicians on subjects which are highly relevant topics and where evidence may be new, limited, or evolving. Expert consensus statements are meant to fill in critical practice gaps when there is insufficient published data to provide the recommendations with the level of evidence expected from guidelines; they are also helpful in synthesizing newer information. Such expert consensus statements, also sometimes presented by some organizations as *expert consensus decision-making pathways*, provide the agreed upon recommendations of a panel of subject matter experts. These recommendations are often based on a combination of systematic review of the available literature, as well as a comprehensive synthesis of existing research, accepted best practice patterns, as well as expert opinion. Such consensus statements could have a clinical or technical focus.

A *position statement* is a document written by an expert panel that addresses an important clinical, technical, safety, or policy issue. The goal is often to provide appropriate background information as well as rationale behind the position adopted. The document can convey recommendations for the society based on limited evidence but on an important evolving topic. The position statement may result from a specific task force designed to review that issue.

*Appropriate use criteria* (AUC) statements provide a framework for clinicians, payers, and hospitals to evaluate the appropriateness of CMR within the context of other cardiovascular testing or services. Unlike guidelines or consensus statements which guide clinical practice, such documents often specify whether it is appropriate, may be appropriate, or rarely appropriate to consider a procedure or test in various scenarios. Such tools are meant to supplement physician clinical judgement and designed to reduce practices for which there is a lack of evidence or benefit. AUC documents are designed to promote quality of care, efficiency, and cost-effectiveness, and may also impact reimbursement. Working groups or special interest groups may include representation of various stakeholders, often including experts from multiple societies.

## Pathways to plan and initiate endorsed papers

There are four pathways to initiate an SCMR endorsed manuscript including: (1) SCMR leadership initiated, (2) SCMR membership initiated, (3) SCMR working group or special interest group initiated, and (4) manuscripts initiated by other societies for SCMR partnership and/or endorsement.

*SCMR leadership initiated papers* SCMR leadership initiated papers are initiated by the leadership of the SCMR (executive committee or board of trustees). The Executive Committee or board of trustees (BOT) can charge the publications committee with identifying experts necessary to write the proposal, or the Executive Committee or BOT could identify the experts themselves.

*SCMR membership initiated papers* Any member of the SCMR can propose a paper. The proposal will be submitted to the Publications Committee as outlined below.

*SCMR working group or special interest group initiated papers* An SCMR working group or special interest group can propose a paper. The proposal will be submitted to the Publications Committee as outlined below.

SCMR may receive manuscripts from other societies for SCMR endorsement or be asked to participate in the joint writing of these manuscripts with other societies. The Executive Committee and BOT determine which manuscripts they would like to participate in and can ask the Publications Committee to review proposals and manuscripts from other societies.

A proposal for an SCMR-endorsed document should meet the following criteria:

- *Length* the proposal should be 1–2 pages in length using Times New Roman 12 pt font size and double spaced text.
- *Title* the title should include the phrases “SCMR” and “recommendations” or “expert consensus”.
- *Main purpose* a statement of the aims, primary purpose, and audience of the proposal.
- *Justification* the intended focus of the manuscript, the rationale for publication, and a confirmation that a brief literature review was performed to establish that paper does not overlap with previous or planned publications.
- *Relevance to SCMR* provide the rationale as to why this proposal should be endorsed by the SCMR. In addition, if the authors aim to seek endorsements from other societies or if this is a collaborative writing project with other societies, please give a rationale and proposed societies.
- *Proposed writing group* The proposed authors should be *acknowledged experts* in the matter of the proposal. The proposal should specify each of the proposed authors, their specialty, and their current institution. Authors should strive for a balance of specialty and geography, assuming that this can be done while only including authors who are acknowledged experts. Specialties of proposed authors should include cardiology, radiology, physics, and technologists when appropriate. In addition, geographic balance should be strived for including authors from North America, Europe, Asia, South America, Africa, and Oceania when possible.
- *Anticipated Timeline* The proposal must contain a timeline with deadlines for submission to the Publication Committee. Failure to meet the accepted timeline allows consideration of competing proposals.
- *Corresponding author* The proposal should designate an author who will liaison with the Publication Committee during wiring of the paper. The corresponding author should include their contact information including email address and phone number.
- *Submission* Proposal should be submitted to the chairperson of the Publications Committee.

## Process

The process of proposal submission, Publications Committee review and ultimate publication in the *JCMR* is summarized in Table 1. The guidelines for engaging with other societies (endorsements of documents, joint writing groups) are summarized below (Sect. “Engagement with Other Societies and Endorsement of Documents”). A completed proposal should be submitted to the Publications Committee chair via email. After review by the Publication Committee and subsequently the Executive Committee, a decision will be returned to the corresponding author with a timeline for submission and periodic updates ranging from 3 to 12 months based on the scope of the proposal. Submitted manuscripts will be sent to reviewers selected by the Publications Committee with comments and required revisions sent to corresponding authors. Revised manuscript will then be re-reviewed by the Publications Committee chair and the Executive Committee with a decision for publication or the need for additional revisions communicated to the corresponding authors. Once a manuscript is deemed satisfactory for publication, the manuscript will be sent directly to the *JCMR*. The manuscript will not undergo additional external peer review at *JCMR*, however it will be subject to review by the *JCMR* editor-in-chief with potential additional revisions requested prior to publication.

**Table 1:** Timeline and process for proposals sent to the publication committee

1. Written proposal submitted to Publications Committee	
2. Proposal reviewed by Publications Committee to determine suitability for SCMR endorsement. The Publications Committee responds to the corresponding author with recommendations	4 weeks
3. Corresponding author responds to the recommendations of the Publications Committee	
4. The Publication Committee reviews the corresponding authors response and if response is acceptable asks the Executive Committee to review the proposal	
5. SCMR Executive Committee review of proposal and recommendation with decision sent to the Publications Committee and the corresponding author. The Executive Committee may suggest SCMR board or Executive Committee members as additional co-authors	4 weeks
6. Writing of guideline, expert consensus, white paper and expert consensus	3-12 months^*^
7. Review by Publication Committee with feedback:	4 weeks
a. Initial version of manuscript reviewed by 3–4 reviewers as selected by the Publications Committee as well as chair of Publications Committee/representing delegate	
b. Reviews of the Publications Committee sent to corresponding authors	
8. Authors revise manuscript and response to Publication Committee	8–12 weeks
9. Resubmission reviewed by the Publications Committee chair. Re-review by external reviewers performed at discretion of Publication Committee chair	4 weeks
10. Feedback from the Publications Committee sent to the Executive Committee for manuscript review	2 weeks
11. Communication with authors regarding manuscript acceptance and/or revisions needed	
12. Final Publication Committee approved manuscript submitted to *JCMR* for review by the editor-in-chief, editorial changes with feedback directly to the author	4 weeks

*Typical 3–6 months for white paper and 6–18 months for guideline or expert consensus.

### Peer review selection

The Publications Committee chair will select 3–5 reviewers to review proposals and manuscripts. The reviewers will generally be chosen from the members of the Publications Committee who have expertise/interest in the topic and field in question. If there are not enough expert reviewers with expertise in the topic in question among the members of the Publications Committee, then the Publication Committee chair can recruit expert reviewers from outside the committee.

## Writing instruction

### Publication structure/template

**Table:** 

	Description
Abstract	As per *JCMR* instructions
Authors	Full list of authors, specialty, country of practice
Introduction/purpose	Define clinical consensus statement and its purpose
Background	Rationale for a consensus statement on the topic, and the literature that currently exists within the topic area
Methods	Methodology behind the literature search and the selection of experts
Results	Highlight the results of the literature search. Discuss key statements that achieved consensus by topic area
Discussion	Overview of the achieved consensus statement
Summary	Summarize manuscript, consensus and clinical implications

### Formatting guidelines

Times New Roman with a 12 pt font size and double line spacing should be used (Table 2).

**Table 2:** Publication structure/template

Type of document	Maximum number of words	Maximum number of tables/figures	Maximum number of references
White paper	3500	5	50–75
Expert consensus statement	3500	5	50–75
Clinical practice guideline	5000–7500	5–15	100–150

## Engagement with other societies and endorsement of documents

### Endorsement of SCMR documents by outside societies

If endorsement by outside societies is sought for any of the documents (Guidelines, Expert Consensus, Recommendations and White Papers), it is recommended that the writing groups follow the process outlined below.

- The writing group submits a request for outside society endorsement(s) to the Publications Committee
- Publications Committee review of the requested outside society endorsement(s) (approve/disapprove), with final approval by the SCMR Executive Committee
- The writing group contacts the societies approved for outside endorsement to receive their process/guidelines for endorsement of SCMR documents
- As necessary, the writing group should include outside societies in the writing and peer-review of the guideline, expert consensus, white paper, and expert consensus document.

### SCMR endorsement of documents developed by outside societies

Documents endorsed by the SCMR represent official SCMR guidance, consensus or recommendations and are reviewed under the same procedures as other SCMR endorsed documents, including internal stakeholder peer review and final approval by the SCMR Executive Committee. For SCMR endorsement of documents developed by outside societies, the following process should be followed.

- SCMR Executive Committee or the Publications Committee choose a writing group member to represent SCMR
- During peer review, SCMR Executive Committee or the publications committee identify an SCMR-designated peer reviewer
- After peer review, the outside organization sends revised document and peer review comments/responses to the SCMR Publication Committee for review and comments (anticipated turnaround time 4 weeks)
- Final approval by Publications Committee and SCMR Executive Committee.
- The document is endorsed by SCMR and link is posted on SCMR website upon publication

Preferably, SCMR representatives or reviewers participate early in the external document development process. Early and ongoing communication regarding document development is optimal. Consistent with SCMR policy, the endorsed document must be free of commercial involvement or bias, whether financial or intellectual.

## Dissemination

During the development phase, a plan should be developed for dissemination of the white paper, expert consensus statement or guidelines and depends on the nature of the document and target audience. Also, a plan to ensure that evaluation and revision will take place is a requirement. Once published, the full-text version of the manuscript should be made freely available to all members of the public.

## Conflict of interest

Before the commencement of writing a manuscript, writing members are asked to declare in writing any interests relevant to this work (see “Appendix”). All declarations are added to a central document which is available throughout the development of the manuscript, allowing members to take potential conflicts of interest into consideration during discussions, writing and decision making. Participants will be asked to update their information throughout the process if there are any changes to the initial declaration. If members are identified as having a significant real or perceived conflict of interest, it could be decided to put conflict of interest precautions or strategies in place. As a result, a person with a significant conflict of interest might be excluded from authorship.

## Figures and Tables

**Table 3:**
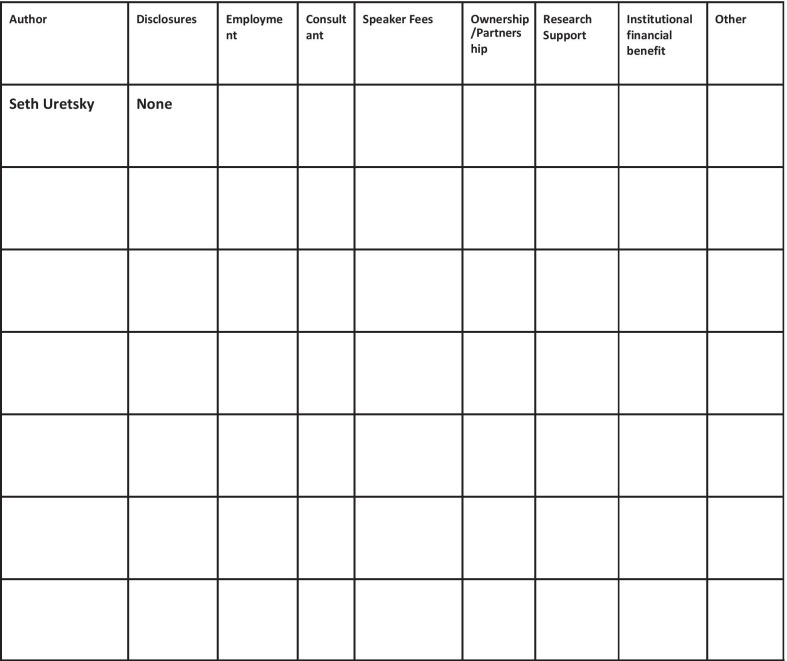
Word, figure and reference guidelines per document

## Appendix

See Table 3.

## Abbreviations

AUC: Appropriate use criteria
BOT: Board of trustees
CMR: Cardiovascular magnetic resonance
CV: Cardiovascular
JCMR: Journal of Cardiovascular Magnetic Resonance
SCMR: Society for Cardiovascular Magnetic Resonance

